# Participation of women in clinical studies of atrial fibrillation in the Northern Netherlands

**DOI:** 10.1007/s12471-024-01887-3

**Published:** 2024-08-06

**Authors:** Neda Khalilian Ekrami, Dawid K. Baron, Emelia J. Benjamin, Bart A. Mulder, Isabelle C. Van Gelder, Michiel Rienstra

**Affiliations:** 1 Department of Cardiology, University Medical Centre Groningen, Groningen, The Netherlands; 2 Department of Medicine, Boston Medical Centre, Boston University Chobanian & Avedisian School of Medicine, Boston, MA USA; 3 Department of Epidemiology, Boston University School of Public Health, Boston, MA USA

**Keywords:** Atrial fibrillation, Women representation, Sex differences, Clinical trials

## Abstract

**Introduction:**

Concerns exist of women underrepresentation in atrial fibrillation (AF) studies, potentially limiting the generalisability of study findings to women with AF. We assessed the participation of women in AF clinical studies performed at a tertiary care centre in the Northern Netherlands.

**Methods:**

Eight AF clinical studies with screening logs were available for analysis. To identify sex-specific differences, patient inclusion and exclusion and reasons for exclusion were assessed. Participation-to-prevalence ratios (PPRs) were calculated to evaluate the representation of women in the studies relative to the AF sex distribution of the general population in the Netherlands (2019 Global Burden of Disease study).

**Results:**

We included 1739 screened patients with AF in the analysis, of whom 722 (41.5%) were women. Of the patients screened, 161 (9%) were enrolled. Median age of screened patients was 69 years (interquartile range (IQR): 61–77), and women were older than men (71 years; IQR: 63–79 vs 68 years; IQR: 60–75; *p* < 0.001). Women were not underscreened compared with men (PPR: 1.09; 95% confidence interval (CI): 1.08–1.10), disproportionally excluded (92% vs 90%; *p* = 0.10) or less willing to participate (17% vs 15%; *p* = 0.36). Women had an overall PPR of 1.05 (95% CI: 1.05–1.06) compared with the general AF population.

**Conclusion:**

At our tertiary hospital in the Northern Netherlands, women appeared to be well-represented in AF studies. The current study advocates for the adoption of a more comprehensive measure of equity, such as the PPR, and screening log evaluation to improve the generalisability of study findings to the entire clinical AF population.

**Supplementary Information:**

The online version of this article (10.1007/s12471-024-01887-3) contains supplementary material, which is available to authorized users.

## What’s new?

- There are concerns of women underrepresentation in clinical studies on atrial fibrillation (AF).
- In the Northern Netherlands, women were well-represented in AF clinical studies and compared with men, they were not underscreened, disproportionally excluded or less willing to participate.
- The current work advocates for the use of equity measures in assessing women representation, such as the participation-to-prevalence ratio (PPR), rather than focusing on strict equality.
- In conjunction with the PPR, the role of screening log evaluation is highlighted in ensuring adequate women representation and improving study generalisability to the entire clinical AF population.

## Introduction

Atrial fibrillation (AF) is the most common sustained arrhythmia encountered in clinical practice, representing a complex and heterogenous entity. The effective management of patients with AF can be challenging. Sex-specific factors influence AF presentation, management, treatment responses and patient outcomes, and it is important to explore these interactions further [1–4]. Sex typically denotes the biological and physiological attributes that differentiate individuals as male or female, while gender is relatively more fluid, encompassing social, cultural and individual factors that define an individual’s sense of being female, male or other. Despite known sex-associated differences, concerns exist that women are underrepresented in AF studies, which may result in findings with uncertain generalisability to women with AF [5–7].

Many post-hoc analyses have reported that women (based on sex differentiation) with AF are often older, have more symptoms and more severe symptoms, and have a lower quality of life than affected men [6, 8, 9]. An increased risk of long-term AF-associated complications such as stroke and heart failure, as well as cardiovascular events (hospitalisation or death) and all-cause mortality, have been described in affected women [6, 10]. Several studies have shown that women have relatively less access to rhythm-control strategies, such as ablation and cardioversion [1, 4, 11, 12]. Whether this difference in treatment approach is due to appropriate clinical judgement, patient preferences and/or treatment inequities warrants further investigation [1–3, 13–15]. Sex-specific differences in treatment-associated side effects have also been reported, including a relatively increased risk of major bleeding in women with AF treated with anticoagulation [16]. Given the interactions between sex and AF, it is crucial that adequate representation of women is attained in clinical studies of AF to ensure that their findings are translatable and generalisable to the clinical setting and improve the effective management and outcomes of all AF patients.

The aim of the current study was to assess the participation of women in AF clinical studies performed at a tertiary care centre in the Northern Netherlands. Sex distribution in these studies was assessed relative to the AF sex distribution of the general population, and reasons for exclusion were evaluated.

## Methods

### Study selection and screening procedure

A total of 10 AF studies were performed at the Department of Cardiology of the University Medical Centre Groningen (UMCG) in Groningen, the Netherlands in the period 2005–2023. For 8 studies, screening logs were available, and these studies were therefore included in the current analysis (see Tables S1 and S2 in Electronic Supplementary Material). All included studies were multicentre in design, but we solely report on UMCG patients. Only prospective studies were assessed, including 7 interventional trials and 1 observational study.

All patients included in the analysis had paroxysmal, persistent or permanent AF. Patients presenting with cardiovascular disease in either the outpatient or emergency room setting were evaluated for AF, which was diagnosed by 12-lead ECG and confirmed by the treating physician. All patients with AF were subsequently placed in the screening log and study-specific inclusion and exclusion criteria were recorded. Eligible patients were invited to participate in the respective study. Reasons for patient exclusion were noted. Patient participation was voluntary, and patients did not receive any reimbursement for involvement. All included patients provided written informed consent, and all included studies were conducted in accordance with the Declaration of Helsinki and approved by the Medical Ethics Committee of the UMCG.

### Data collection

Patient inclusion and exclusion were assessed using screening logs. To identify sex-specific differences in participation, reasons for exclusion were evaluated (by D.K.B. and M.R.) and further subcategorised into 6 groups: inclusion criteria not met or exclusion criteria present, logistical reason, physician preference, patient preference, other and unknown (see Tables S3 and S4 in Electronic Supplementary Material). Study participation was also assessed following subcategorisation of patients with AF by setting (outpatient vs emergency room) and study type (industry-sponsored vs investigator-initiated).

At the time of data collection, only sex information was attained and gender information was not available for the included studies. The current work therefore only focusses on sex-specific differences.

### Participation-to-prevalence ratio

The participation-to-prevalence ratio (PPR) was used to describe the representation of women in the AF studies relative to the sex distribution of AF patients in the general population. The PPR was calculated by dividing the percentage of women among study participants by the percentage of women with AF in the general population. A PPR of 0.8–1.2 indicates the sex distribution in the studies approximated that of the general population, whereas a PPR < 0.8 or > 1.2 indicates sex underrepresentation and overrepresentation, respectively [5, 17–20].

The sex distribution of AF in the general population in the Netherlands was obtained from the Global Burden of Disease (GBD) study database [21]. In 2019, a total of 274,110 Dutch patients had an AF diagnosis: 108,541 women (40%) and 165,569 men (60%). The total sex-stratified and age-stratified AF prevalence data of the GBD study are included in Table S5 in the Electronic Supplementary Material. Calculations were also performed to assess the representation of women in the studies compared with that of AF patients at the UMCG. In the period 2018–2022, 29,000 UMCG patients had an AF diagnosis: 11,112 women (38%) and 17,888 men (62%).

### Statistical analysis

Descriptive outcomes are presented as number (%) or median with interquartile range (IQR), and sex representation is presented as PPR with 95% confidence interval (CI). Differences between groups were analysed with the chi-squared test for categorical data. A two-sided *p*-value ≤ 0.05 was considered to be statistically significant. Analyses were performed using SPSS 28 software (Chicago, IL, USA).

## Results

### Patient characteristics during screening and study participation

A summary of the patients characteristics is shown in Tab. 1. A total of 1739 screened patients with AF were included in the current analysis: 722 women (41.5%) and 1017 men (58.5%) (see Table S2 in Electronic Supplementary Material). Median age was 69 years (IQR: 61–77). Women were significantly older than men (71 years; IQR: 63–79 vs 68 years; IQR: 60–75; *p* < 0.001) (Tab. 2). After screening, 1578 patients (91%) were excluded from participating, of whom 665 women (42%) and 913 men (58%). No significant association was found between sex and study inclusion (8% of women vs 10% of men; *p* = 0.10). For both sexes, the most common reason for exclusion was that the inclusion criteria were not met or an exclusion criterion was present. No significant sex differences were found in any of the exclusion reason categories, including patient willingness (‘patient preference’).

**Table 1:** Frequencies of included and excluded patients screened for participating in AF studies and reasons for exclusion, stratified by sex

Variable	Patients (*N* = 1739)	Women (*n* = 722)	Men (*n* = 1017)	*P*-value
Inclusion	161 (9)	57 (8)	104 (10)	
Exclusion	1578 (91)	665 (92)	913 (90)	0.10
*Reason for exclusion*^*a*^				
Inclusion criteria not met or exclusion criteria present	1186 (75)	496 (75)	690 (76)	0.65
Logistical reason	77 (5)	25 (4)	52 (6)	0.08
Physician preference	8 (0)	3 (0)	5 (1)	1.00
Patient preference	248 (17)	111 (17)	137 (15)	0.36
Other	7 (0)	3 (0)	4 (0)	1.00
Unknown	44 (3)	23 (3)	21 (2)	0.17
Deceased	8 (0)	4 (1)	4 (0)	0.73

Data are *n* (%)

*AF* atrial fibrillation

^a^Percentages are relative to number of excluded patients

**Table 2:** Age distribution of patients screened for participating in AF studies and associated PPRs, stratified by sex

Age in years	Patients (*N* = 1739)	Women (*n* = 722)	Men (*n* = 1017)	*P*-value^a^	PPR in women^b^	PPR in men^b^
< 40	47 (3)	14 (2)	33 (3)	0.10	*2.01*	0.82
40–49	86 (5)	34 (5)	52 (5)	0.70	*2.28*	*0.73*
50–59	263 (15)	93 (13)	170 (17)	* 0.03*	*1.56*	0.84
60–69	502 (29)	181 (25)	321 (32)	* 0.003*	1.16	0.93
70–79	553 (32)	245 (34)	308 (30)	0.10	1.09	0.94
80–89	270 (15)	145 (20)	125 (12)	*<* *0.001*	1.02	0.98
> 90	18 (1)	10 (1)	8 (1)	0.22	0.84	*1.32*

Data are *n* (%) or participation-to-prevalence ratio (PPR)

*AF* atrial fibrillation

^a^*P*-value ≤ 0.05 is marked in italic

^b^PPRs in italic are outside range of 0.80–1.20, indicating sex distribution in AF studies did not approximate that of AF patients in general population

However, there was a significant association between sex and setting type (*p* = 0.04; see Table S6 in Electronic Supplementary Material). Women were more often screened for studies in an emergency room setting than men, while men were more often screened for outpatient studies. No significant association between sex and study type was found (*p* = 0.64).

### Women representation

Investigation of the women representation in the included AF studies relative to the sex distribution of AF patients in the general population showed that women had an overall PPR of 1.05 (95% CI: 1.05–1.06), and similar ratios were observed after subcategorisation by setting and study type (Tab. 3). Men were found to have a relatively lower overall PPR (0.98; 95% CI: 0.98–0.98). Following age stratification, women had a relatively higher PPR at younger ages, which decreased with increasing age. Conversely, men had a relatively lower PPR at younger age that increased with increasing age (Tab. 2).

**Table 3:** PPR of patients in AF studies relative to AF patients in the general population and in the UMCG, stratified by sex

Variable	Women	Men
*Study to general population*	1.05 (1.05–1.06)	0.98 (0.98–0.98)
Outpatient setting	1.00 (0.99–1.00)	1.02 (1.01–1.02)
Emergency room setting	1.12 (1.11–1.13)	0.92 (0.92–0.93)
Industry-sponsored studies	1.08 (1.07–1.09)	0.98 (0.97–0.99)
Investigator-initiated studies	1.04 (1.04–1.04)	0.98 (0.97–0.98)
*Study to UMCG population*	1.09 (1.08–1.10)	0.95 (0.95–0.95)
Outpatient setting	1.03 (1.03–1.04)	0.98 (0.98–0.99)
Emergency room setting	1.16 (1.15–1.16)	0.90 (0.90–0.91)
Industry-sponsored studies	1.12 (1.10–1.13)	0.94 (0.93–0.95)
Investigator-initiated studies	1.07 (1.07–1.08)	0.96 (0.95–0.96)
UMCG to general population	0.97	1.02

Data are participation-to-prevalence ratio (PPR) (95% confidence interval)

*AF* atrial fibrillation, *UMCG* University Medical Centre Groningen

In the AF patient population at the UMCG, women and men had a PPR of 0.97 and 1.02, respectively, compared with the general population. The representation of both women (PPR: 1.09; 95% CI: 1.08–1.10) and men (PPR: 0.95; 95% CI: 0.95–0.95) in the included studies approximated that of the entire UMCG AF population.

## Discussion

The aim of the current study was to assess the participation of women in AF clinical studies at a tertiary care centre. At the UMCG, women were found to be well-represented in the AF studies with available screening logs, and they did not appear to be underscreened. There was no significant association between sex and study inclusion or any of the evaluated reasons for exclusion, including patient willingness. The current work advocates for the adoption of a more comprehensive and nuanced measure of equity, such as the PPR, in conjunction with screening log evaluation to ensure equitable access to study participation for women and men, and to improve the generalisability of study findings to the entire clinical AF population.

For clinical studies that aim to influence the real-world patient population, it is important to focus on equity rather than strict equality. Sex-specific differences in disease prevalence, presentation and treatment responses exist, and accounting for these differences ensures a more accurate representation of real-world diversity and that study findings are both effective and safe for the patient population. Conversely, findings from clinical studies with significant deviations in patient proportions from those of the clinical population will likely fail to adequately inform medical practice, hinder appropriate healthcare decision-making and may pose risks or harm to one particular group [22–24]. While adequate representation in the clinical population is desirable, it poses logistical challenges that can complicate and lengthen the recruitment process and impede study progression.

Previous studies assessing women’s representation in AF clinical trials report PPRs ranging from 0.8 to 1.2 (see Table S7 in Electronic Supplementary Material) [5, 17, 19, 25, 26]. Based on these studies, women do not appear to be underrepresented in AF clinical studies, which is in accordance with the findings of the current work. Jin et al. reported a PPR for women representation of 0.78 in cardiovascular clinical trials relating to arrhythmia, although this category was not restricted to AF alone and also included other arrhythmias that vary in their sex-disaggregated prevalence [18].

In the current work, both women and men had adequate representations in the analysed trials. Women were found to have an adequate representation during the screening phase of the available AF studies compared with the sex distribution at the UMCG, and study inclusion was not found to be significantly associated with sex, suggesting that women were neither disproportionally underscreened nor excluded from participating in AF studies. At screening, women were older than men, which is in accordance with other studies reporting women with AF tended to be older upon diagnosis [6, 8, 22–24]. Following stratification by setting type, women were more often screened for emergency room studies than men, whereas men were more frequently screened for outpatient studies. This finding may be partially explained by the relatively later onset and higher severity of AF symptoms in women, which may necessitate more immediate care. Alternatively, it may suggest that women are less likely to receive an early diagnosis, but the exact mechanism behind this finding calls for further investigation. Several studies have reported a relatively decreased willingness to participate in studies among women [18, 27–29]. Interestingly, this was not a finding of the current work.

In further investigating women participation, screening logs will become invaluable assets for assessing demographic representation and ensuring quality control in the recruitment process. In addition to utilising screening logs, PPR calculations may be performed in the planning stages of a clinical study to set targets with the aim of attaining appropriate patient representation. Together, these steps can improve the generalisability of study findings and subsequently the management and outcomes of all patients with AF.

### Limitations

Several limitations in the current work require consideration. First, the included studies were performed at a tertiary care centre, and the findings may not be applicable to other care settings. Additionally, the number of patients with AF who did not meet study inclusion criteria was substantial, and the study population represented a highly selected AF population, potentially limiting the generalisability of the study findings to the real world.

Second, 2 of the 8 included studies (INSTANT, ClinicalTrials.gov Identifier: NCT03539302; Cardiome - RSD1235-SR: NCT00267930) had an upper age limit for inclusion of 85 years, which may disproportionally exclude women from participating due to the later onset of disease. Third, calculation of the PPR is influenced by the sex distribution of the disease in the general population, which may be difficult to estimate and may show variability among sources. Therefore, the reported PPRs may not be generalisable to other populations that differ significantly from the studied population. Fourth, the available studies only collected information on patient sex and not gender, which may influence patient engagement and representation in clinical trials.

Last, screening log utilisation by treating physicians was not strictly regulated, and physicians had freedom in the amount of information that they provided in the screening logs. As a result, not all screening log information was uniform and complete. The available information allowed for the broad categorisation of reasons for exclusion, but more subtle differences that impact women participation may have been missed. A more systematic approach to screening log utilisation is recommended for future analyses of patient trial participation trends.

## Conclusion

The current study contributes to the ongoing dialogue on the representation of women in AF clinical studies and provides valuable insights into women participation. Contrary to prevailing belief, the current work and other previous studies report that women are well-represented in AF studies. It is imperative that researchers and regulatory bodies prioritise the comprehensive evaluation of screening logs and patient representation to discern and rectify any disparities, barriers to participation, biases and systemic issues influencing patient participation in AF clinical studies. It is also important that we continue to explore how AF intersects with other sociodemographic factors beyond sex [30], advance efforts that strive to promote diversity, develop evidence-based interventions that are safe and effective and make policies that create more inclusive and equitable research environments.

### Supplementary Information

Tables S1–S7: Analyzed study and sex distribution information
