# Supplementary material for: Participation of women in clinical studies of atrial fibrillation in the Northern Netherlands
Source: Neth Heart J. 2024 Aug 6;32(9):326–31. doi: 10.1007/s12471-024-01887-3 (PMC11335698; doi:10.1007/s12471-024-01887-3)
Supplement: Supplementary file 1 — Tables S1–S7: Analyzed study and sex distribution information [file 12471_2024_1887_MOESM1_ESM.docx]

**SUPPLEMENTARY INFORMATION**

**Table S1. Studies included within the analysis**

| **Clinical Study** | **Setting type** | **Study type** | **ClinicalTrials.gov Identifier** |
| --- | --- | --- | --- |
| RE-LY | outpatient | industry-sponsored | NCT00262600 |
| Cardiome - RSD1235-SR | outpatient | industry-sponsored | NCT00267930 |
| AXAFA-AFNET 5 | outpatient | investigator-initiated | NCT02227550 |
| RACE 4 | outpatient | investigator-initiated | NCT01740037 |
| RACE V | outpatient | investigator-initiated | NCT02726698 |
| INSTANT | emergency room | industry-sponsored | NCT03539302 |
| RACE 7 ACWAS | emergency room | investigator-initiated | NCT02248753 |
| RACE 9 OBSERVE-AF | emergency room | investigator-initiated | NCT04612335 |

AF, atrial fibrillation; AXAFA-AFNET 5, Anticoagulation using the direct factor Xa inhibitor apixaban during Atrial Fibrillation catheter Ablation: comparison to VKA therapy; Cardiome - RSD1235-SR, A Pilot Phase II, Randomised, Double-blind, Placebo-controlled, Multi-centred Safety, Tolerability and Preliminary Efficacy Study of RSD1235-SR for the Prevention of Atrial Fibrillation/Atrial Flutter (AF/AFL) Recurrence in subjects Post-Conversion from AF; INSTANT, INhalation of flecainide to convert recent onset SympTomatic Atrial fibrillation to siNus rhyThm; RACE 4, integRAted Chronic Care program at specialized AF Clinic Versus usual CarE in patients with atrial fibrillation; RACE V, Reappraisal of Atrial fibrillation: interaction between hyperCoagulability, Electrical remodeling, and vascular destabilisation in the progression of atrial fibrillation; RACE 7 ACWAS, RAte Control versus Electrical cardioversion trial 7–Acute Cardioversion versus Wait And See; RACE 9 OBSERVE-AF, device-based rate versus rhythm control treatment in patients with symptomatic recent-onset atrial fibrillation in the emergency department; RE-LY, Randomized Evaluation of Long-term anticoagulation therapY.

**Table S2. AF study population counts stratified by study**

| **Clinical Study** | **Total AF** | **Women AF** | **Men AF** |
| --- | --- | --- | --- |
| RE-LY | 262 | 99 | 163 |
| Cardiome - RSD1235-SR | 17 | 5 | 12 |
| AXAFA-AFNET 5 | 14 | 5 | 9 |
| RACE 4 | 59 | 22 | 37 |
| RACE V | 628 | 255 | 373 |
| INSTANT | 348 | 161 | 187 |
| RACE 7 ACWAS | 79 | 36 | 43 |
| RACE 9 OBSERVE-AF | 332 | 139 | 193 |
| **Total** | **1739** | **722** (41.5%) | **1017** (58.5%) |

AF, atrial fibrillation; AXAFA-AFNET 5, Anticoagulation using the direct factor Xa inhibitor apixaban during Atrial Fibrillation catheter Ablation: comparison to VKA therapy; Cardiome - RSD1235-SR, A Pilot Phase II, Randomised, Double-blind, Placebo-controlled, Multi-centred Safety, Tolerability and Preliminary Efficacy Study of RSD1235-SR for the Prevention of Atrial Fibrillation/Atrial Flutter (AF/AFL) Recurrence in subjects Post-Conversion from AF; INSTANT, INhalation of flecainide to convert recent onset SympTomatic Atrial fibrillation to siNus rhyThm; RACE 4, integRAted Chronic Care program at specialized AF Clinic Versus usual CarE in patients with atrial fibrillation; RACE V, Reappraisal of Atrial fibrillation: interaction between hyperCoagulability, Electrical remodeling, and vascular destabilisation in the progression of atrial fibrillation; RACE 7 ACWAS, RAte Control versus Electrical cardioversion trial 7–Acute Cardioversion versus Wait And See; RACE 9 OBSERVE-AF, device-based rate versus rhythm control treatment in patients with symptomatic recent-onset atrial fibrillation in the emergency department; RE-LY, Randomized Evaluation of Long-term anticoagulation therapY.

**Table S3. Reasons for study non-inclusion categories and frequencies**

|  | **Total AF non-inclusion**  (n = 1578) | **Women AF non-inclusion**  (n = 665) | **Men AF non-inclusion**  (n = 913) |
| --- | --- | --- | --- |
| **Inclusion criteria not met** | 1186 (75%) | 496 (75%) | 690 (76%) |
| **Logistic reasons** | 77 (5%) | 25 (4%) | 52 (6%) |
| - patient was already participating in another study | 24 (2%) | 3 (0%) | 21 (2%) |
| - patient was admitted outside working hours | 22 (1%) | 9 (1%) | 13 (1%) |
| - scheduling difficulties | 4 (0%) | 3 (1%) | 1 (0%) |
| - technical difficulties and/or malfunctions with screening/inclusion | 10 (1%) | 5 (1%) | 5 (1%) |
| - language barrier | 6 (0%) | 1 (0%) | 5 (1%) |
| - the research site was too far and/or the patient did not have adequate access to transportation | 4 (0%) | 1 (0%) | 3 (0%) |
| - patient did not possess a smartphone and/or access to e-mail | 7 (1%) | 3 (1%) | 4 (1%) |
| **Physician preference** | 8 (0%) | 3 (0%) | 5 (1%) |
| **Patient preference** | 248 (17%) | 111 (17%) | 137 (15%) |
| - patient did not wish to be informed about the study | 8 (1%) | 6 (1%) | 2 (0%) |
| - patient did not wish to participate in the study | 156 (10%) | 63 (10%) | 93 (10%) |
| - patient was “too busy” | 3 (0%) | 0 (0%) | 3 (0%) |
| - patient found the study too intensive and/or long in duration | 35 (3%) | 17 (3%) | 18 (2%) |
| - patient did not wish to alter their current treatment strategy and/or was set on receiving a specific treatment | 24 (2%) | 12 (2%) | 12 (2%) |
| - patient did not wish to receive an implantable-loop recorder | 16 (1%) | 9 (1%) | 7 (1%) |
| - patient was anxious about participating | 3 (0%) | 2 (0%) | 1 (0%) |
| - patient had negative experiences with previous study participation | 3 (0%) | 2 (0%) | 1 (0%) |
| **Other** | 7 (0%) | 3 (0%) | 4 (0%) |
| - patient exhibited cognitive impairments | 1 (0%) | 0 (0%) | 1 (0%) |
| - patient was in an emergency situation | 3 (0%) | 0 (0%) | 3 (0%) |
| - patient was unavailable due to a concomitant cancer diagnosis | 2 (0%) | 2 (0%) | 0 (0%) |
| - patient was housebound | 1 (0%) | 1 (0%) | 0 (0%) |
| **Unknown** | 44 (3%) | 23 (3%) | 21 (2%) |
| **Deceased** | 8 (0%) | 4 (1%) | 4 (0%) |

Data are presented as number of patients (%).

**Table S4. Inclusion and exclusion criteria of the included AF studies**

| **Randomized Evaluation of Long Term Anticoagulant Therapy (RE-LY) With Dabigatran Etexilate** (NCT00262600) |
| --- |
| **Age:** 18+ years  **Inclusion criteria:**  Patients with non-valvular atrial fibrillation (AF), at moderate to high risk of stroke, or systemic embolism with at least one additional risk factor (i.e. previous ischemic stroke, TIA, or systemic embolism, left ventricular dysfunction, age >=75 years, age >=65 with either diabetes mellitus, history of coronary artery disease or hypertension)  **Exclusion criteria:**   - Prosthetic heart valves requiring anticoagulation, or hemodynamically relevant valve disease that is expected to require surgical intervention during the course of the study - Severe, disabling stroke within the previous 6 months, or any stroke within the previous 14 days - Conditions associated with an increased risk of bleeding - Contraindication to warfarin treatment - Reversible causes of atrial fibrillation (e.g., cardiac surgery, pulmonary embolism, untreated hyperthyroidism). - Plan to perform a pulmonary vein ablation or surgery for cure of the AF - Severe renal impairment (estimated creatinine clearance <=30 mL/min) - Active infective endocarditis - Active liver disease - Women who are pregnant, lactating, or of childbearing potential who refuse to use a medically acceptable form of contraception throughout the study - Anaemia (haemoglobin <100g/L) or thrombocytopenia (platelet count <100 x 109/L) - Patients who have developed transaminase elevations upon exposure to ximelagatran - Patients who have received an investigational drug in the past 30 days - Patients considered unreliable by the investigator concerning the requirements for follow-up during the study and/or compliance with study drug administration, has a life expectancy less than the expected duration of the trial due to concomitant disease, or has any condition which in the opinion of the investigator, would not allow safe participation in the study (e.g., drug addiction, alcohol abuse) - Any known hypersensitivity to galactose if the warfarin used contains galactose |
| **A Pilot Phase II, Randomised, Double-blind, Placebo-controlled, Multi-centred Safety, Tolerability and Preliminary Efficacy Study of RSD1235-SR for the Prevention of Atrial Fibrillation/Atrial Flutter (AF/AFL) Recurrence in subjects Post-Conversion from AF (Cardiome - RSD1235-SR)** (NCT00267930) |
| **Age:** 18 – 85 years  **Inclusion criteria:**   - Subjects must have sustained, symptomatic atrial fibrillation for greater than 72 hours and less than 6 months duration - Subjects must have adequate anticoagulant therapy   **Exclusion criteria:**   - Subjects may not have Class III or Class IV congestive heart failure - Subjects may not have uncorrected electrolyte imbalance |
| **Apixaban During Atrial Fibrillation Catheter Ablation: Comparison to Vitamin K Antagonist Therapy (AXAFA)** (NCT02227550) |
| **Age:** 18+ years  **Inclusion criteria:**   - Non-valvular AF (ECG-documented) with a clinical indication for catheter ablation - Clinical indication to undergo catheter ablation on continuous anticoagulant therapy - Presence of at least one of the CHADS2 stroke risk factors:   - Stroke or TIA  - age ≥ 75 years  - hypertension, defined as chronic treatment for hypertension, estimated need for continuous antihypertensive therapy or resting blood pressure > 145/90 mmHg  - diabetes mellitus  - symptomatic heart failure (NYHA ≥ II)  **Exclusion criteria:**   - Any disease that limits life expectancy to less than 1 year - Pregnant women or women of childbearing potential not on adequate birth control: only women with a highly effective method of contraception (oral contraception or intra-uterine device) or sterile women can be randomized - Breastfeeding women - Drug abuse or clinically manifest alcohol abuse - Any stroke within 14 days before randomization - Coadministration with drugs that are strong dual inhibitors of cytochrome P450 3A4 (CYP3A4) and P-glycoprotein (P-gp) or strong dual inducers of CYP3A4 and P-gp (Appendix VIII) - Valvular AF (as defined by the focused update of the ESC guidelines on AF, i.e. severe mitral valve stenosis, mechanical heart valve). Furthermore, patients who underwent mitral valve repair are not eligible for AXAFA - Any previous ablation or surgical therapy for AF - Cardiac ablation therapy for any indication (catheter-based or surgical) within 3 months prior to randomization - Clinical need for "triple therapy" (combination therapy of clopidogrel, acetylsalicylic acid, and oral anticoagulation) - Other contraindications for use of VKA or apixaban - Documented atrial thrombi less than 3 months prior to randomization - Severe chronic kidney disease with an estimated glomerular filtration rate (GFR) < 15 ml/min |
| **IntegRAted Chronic Care Program at Specialized AF Clinic Versus Usual CarE in Patients With Atrial Fibrillation (RACE4) (**NCT01740037) |
| **Age:** 18+ years  **Inclusion criteria:**   - Patients with newly diagnosed AF detected on electrocardiogram (ECG), Holter recordings or event recorder with a duration > 30 seconds, 3 months before inclusion, or - Patients with a history of diagnosed AF, with no regular control at a cardiologist for AF in the last 2 years and referred by a (non-)cardiologic medical specialist for new diagnostics or therapeutic issue   **Exclusion criteria:**   - No electrocardiographic objectified AF - Unstable heart failure defined as NYHA IV or heart failure necessitating hospital admission < 3 months before inclusion - Acute coronary syndrome (acute myocardial infarction or instable angina pectoris, with two of the following characteristics: chest pain and/ or ischemic electrocardiographic changes, and/ or cardiac enzyme rise) < 3 months before inclusion - Untreated hyperthyroidism or < 3 months euthyroidism before inclusion - Foreseen pacemaker, internal cardioverter defibrillator, and/ or cardiac resynchronization therapy - Cardiac surgery ≤ 3 months before inclusion - Planned cardiac surgery - Regular control and treatment, also for AF, at another specialized outpatient cardiac clinic |
| **Reappraisal of Atrial Fibrillation: Interaction Between HyperCoagulability, Electrical Remodeling, and Vascular Destabilisation in the Progression of Atrial Fibrillation (RACE V)** (NCT02726698) |
| **Age:** 18+ years  **Inclusion criteria:**   - New onset or history of paroxysmal, self-terminating AF documented as:   - AF on ECG or Holter-recording or loop recorder  - Subclinical AF detected in implantable cardiac devices (atrial read > 190 beats per minute, lasting > 6 minutes)   - Prior history of self-terminating AF is allowed - Able to undergo implantation of an implantable loop recorder (in patients without a CIED) - In patients already on oral anticoagulation drugs, the following inclusion criteria need to be met (to allow for safe temporary interruption of anti-thrombotic treatment for coagulation phenotyping):   - CHA2DS2-VASc score ≤5 (history of Congestive heart failure, Hypertension, Age ≥ 75 years (doubled), Diabetes mellitus, Stroke/transient ischemic attack (doubled), Vascular disease, Age 65-75 years, female Sex)  - No other indication for oral anticoagulation (e.g. mechanical valve prosthesis)  - Patient is willing to temporarily stop oral anticoagulation drugs (OAC)  **Exclusion criteria:**   - Non-self-terminating, persistent AF - Deemed unsuitable to undergo implantation of loop recorder (in patients without a CIED) - Refusing to temporarily stop OAC for coagulation phenotyping (in patients already on OAC before inclusion in this study) - On waiting list for pulmonary vein isolation or expected to be placed on waiting list within one year - Expected to start with amiodarone - Pregnancy - Life expectancy of less than 2.5 years |
| **INhalation of Flecainide to Convert Recent Onset SympTomatic Atrial Fibrillation to siNus rhyThm (INSTANT) (INSTANT)** (NCT03539302) |
| **Age:** 18 – 85 years  **Inclusion criteria:**   - Subjects with recent-onset symptomatic AF at presentation, with a duration at onset of symptoms from 1 hour to 48 hours, and from one of the following categories:   - First detected episode of paroxysmal AF  - Recurrent episode of paroxysmal AF  - Episode post-cardiac ablation for paroxysmal AF   - Subjects who are prescribed a pill-in-the-pocket regimen (flecainide or propafenone) for paroxysmal AF, or are within 3 months of having undergone ablation of paroxysmal AF, or have experienced an episode of new AF but are not currently experiencing an episode of recent-onset paroxysmal AF, or are known to have paroxysmal AF (or previously diagnosed with paroxysmal AF) and have one or more previous symptomatic episodes but are not currently experiencing an episode of recent-onset paroxysmal AF may consent to pre-study screening prior to presenting with recent-onset symptomatic AF. These subjects will be eligible to receive study drug only when presenting with symptomatic paroxysmal AF of recent-onset (i.e., ≤ 48 hours), consenting to the full study, and after meeting all eligibility criteria   **Exclusion criteria:**   - Hemodynamic and/or cardiac instability, with systolic blood pressure < 100 mmHg or > 150 mmHg, and/or ventricular heart rate < 80 bpm or > 150 bpm. For subjects to meet eligibility criteria, at least 2 of the 3 measurements of vital signs during screening (45, 30, and/or 15 minutes prior to dosing) must meet criteria - Current AF episode treated with Class I or Class III antiarrhythmic drugs or electrical cardioversion. Subjects whose current AF episode has been treated with flecainide are eligible if their total cumulative exposure to flecainide (including the study drug to be administered in this study) does not exceed 320 mg within a 24-hour period, per site standard of care - History of acute decompensated heart failure (HF) - History within 6 months prior to screening of, or present HF with a left ventricular ejection fraction (LVEF) < 45%, and/or Class II or higher HF as defined by the New York Heart Association (NYHA), and/or medication history suggestive of HF, in the opinion of the Investigator. An echocardiogram with LVEF within 6 months of screening is required to demonstrate eligibility. If no echocardiogram is available, subject must undergo a diagnostic echocardiogram using a portable handheld ultrasound device (handheld echocardiogram; HHE) during screening to confirm eligibility - Evidence of current ongoing myocardial ischemia, such as signs (e.g., significant [e.g., > 2 mm] ST segment elevation or depression on ECG, echocardiographic findings suggestive of acute myocardial infarction), symptoms (e.g., angina pectoris, atypical angina pectoris), and/or being medicated with anti-anginal medication. In addition, subjects with signs of prior myocardial infarction (such as pathological Q waves) who are also taking concomitant medications for angina pectoris should be evaluated for presence of ongoing ischemia - History of myocardial infarction (MI) within 3 months of screening - Known uncorrected severe aortic or mitral stenosis - Hypertrophic cardiomyopathy with outflow tract obstruction - Current diagnosis of persistent AF - One or more episodes of atrial flutter within 6 months prior to screening or atrial flutter at presentation - History of any of the following heart abnormalities:   - Long QT syndrome  - Conduction disease (e.g. second- or third- degree heart block, bundle branch block)  - Diagnosed with sinus node dysfunction (e.g., sick sinus syndrome) and/or one of the following:  - (i) history of unexplained or cardiovascular syncope, (ii) known bradycardia suggestive of sinus node dysfunction, and/or (iii) prior electrical or  pharmacological cardioversion associated with prolonged sinus or ventricular pause (e.g., >3 seconds) and/or slow ventricular rhythm (e.g., <45 bpm) at time of conversion Note: Sinus node dysfunction in AF is more prevalent in subjects >75 years old. d) Brugada Syndrome e) Torsades de  pointes (TdP)   - Any of the following ECG-related features:   - QTc interval >480 msec at screening (estimated by the Fridericia's formula)  - QRS duration ≥ 120 ms or history of previous documented wide QRS tachycardia  - Predominantly (i.e., >30%) paced heart rhythm  - Ventricular tachycardia (VT, sustained or non-sustained), or excessive premature ventricular complexes (PVCs, > 20 multifocal PVCs per hour), prior to dosing as per site telemetry. Site telemetry should be equipped with an alarm system for VT and PVCs or be continuously visually observed prior to dosing   - Severe renal impairment (eGFR < 30 mL/min/1.73 m2) or on dialysis - Known abnormal liver function prior to randomization/allocation (including hepatic disease or biochemical evidence of significant liver derangement known prior to randomization/allocation) - Uncorrected hypokalemia (defined as serum potassium <3.6 mEq/L) at screening. If serum potassium result is <3.8 mEq/L at screening, therapeutic correction (e.g., potassium supplementation) is strongly encouraged, although reassessing the serum potassium level is not required as long as a value ≥ 3.6 mEq/L is documented at screening - Subjects with established pulmonary disease in need of inhalation medication. Subjects with COPD are excluded. Subjects with mild to moderate asthma that are not experiencing active symptoms at screening and whose asthma is well controlled with steroids and/or as-needed administration of a bronchodilator are eligible for the study - Known hypersensitivity to flecainide acetate or any of its active metabolites - Concomitant therapy with systemic drugs that are strong inhibitors of CYP 2D6 (e.g. antidepressants, neuroleptics, ritonavir, some antihistamines) or CYP 2D6 inducers (e.g. phenytoin, phenobarbital, carbamazepine) - Treatment with Class I or Class III antiarrhythmic drugs within the last week. Subjects whose current AF episode has been treated with flecainide are eligible if their total cumulative exposure to flecainide (including the study drug to be administered in this study) does not exceed 320 mg within a 24-hour period, per site standard of care - Treatment with amiodarone within the last 12 weeks - Subject is deemed unsustainable for the trial by the Investigator (including but not limited to: patients who are considered at high risk for stroke based on screening coagulation panel or medical history (e.g., CHA2DS2-VASc score); patients with congenital heart disease; patients with history of AF refractory to pharmacological or electrical cardioversion; patients whose AF is secondary to electrolyte imbalance, thyroid disease, or other reversible or non-cardiovascular cause; patients with episodes of syncope; patients with any serious or life threatening medical condition; patients with any acute infection). The subject may be deemed unsuitable for the trial by the Investigator if the subject is not able or willing to inhale the study drug - Known drug or alcohol dependence within the past 12 months as judged by the Investigator - A body mass index > 40 kg/m^2^ - Previous randomization/allocation in this study or treatment with any other investigational drug within 30 days from screening or 5 half-lives of the drug, whichever is longer - Female of childbearing potential who are not surgically sterile, or post-menopausal (defined as no menses for 2 years without an alternative cause), or for whom a negative pregnancy test is unavailable before study entry, or who are pregnant or breast feeding at study entry - Previous administration of flecainide for an episode of paroxysmal AF or new AF did not result in conversion of AF to SR (i.e., subject is considered a non-responder to flecainide) - Cardiac surgery for any of the exclusionary conditions (e.g., valvular disease, hypertrophy, coronary artery disease [CAD], etc.) within the last 6 months prior to screening - Respiratory rate of > 22 breaths per minute |
| **Acute Cardioversion Versus Wait And See-approach for Symptomatic Atrial Fibrillation in the Emergency Department (RACE 7 ACWAS)** (NCT02248753) |
| **Age:** 18+ years  **Inclusion criteria:**   - ECG with atrial fibrillation at the emergency department - Heart rate > 70bpm - Symptoms most probable due to atrial fibrillation - Duration of symptoms < 36 hours - Able to use MyDiagnostick   **Exclusion criteria:**   - Signs of myocardial infarction on ECG - Hemodynamic instability (systolic blood pressure < 100mm Hg, heart rate > 170 bpm) - Presence of pre-excitation syndrome - History of Sick Sinus Syndrome - History of unexplained syncope - History of persistent AF (episode of AF lasting more than 48 hours) - Acute heart failure - Deemed unsuitable for participation by attending physician |
| **Device-based Rate Versus Rhythm Control in Symptomatic Recent-onset Atrial Fibrillation (RACE 9 OBSERVE-AF)** (NCT04612335) |
| **Age:** 18+ years  **Inclusion criteria:**   - ECG with atrial fibrillation - Duration of the current AF episode <36 hours - Symptoms due to atrial fibrillation - Able to use telemetric rhythm recorder   **Exclusion criteria:**   - History of persistent AF (episode of AF lasting more than 48 hours and terminated by cardioversion) - Deemed unsuitable for participation by attending physician - Hemodynamic instability (heart rate >170 bpm, systolic blood pressure <100 mmHg) - Acute heart failure - Signs of myocardial infarction - History of syncope of unexplained origin - History of untreated Sick Sinus Syndrome - History of untreated Wolff-Parkinson-White syndrome |

**Table S5. Age-stratified prevalence of AF in the Netherlands according to the 2019 Global Burden of Disease (GBD) study database**

| **Age** (years) | **Total AF**  (n = 274110.08) | **Women AF**  (n = 108540.99) | **Men AF**  (n = 165569.10) |
| --- | --- | --- | --- |
| < 40 | 545.64 | 80.86 | 464.78 |
| 40 – 49 | 5630.30 | 978.38 | 4651.92 |
| 50 – 59 | 27088.95 | 6137.37 | 20951.58 |
| 60 – 69 | 70011.29 | 21747.77 | 48263.51 |
| 70 – 79 | 100505.98 | 40936.46 | 59569.53 |
| 80 – 89 | 58349.83 | 30723.22 | 27626.62 |
| > 90 | 11978.09 | 7936.93 | 4041.16 |

Data are presented as number of patients.

AF, atrial fibrillation.

**Table S6. AF population frequencies stratified by setting and study type**

|  | **Total AF** (n = 1739) | **Women AF** (n = 722) | **Men AF** (n = 1017) | **P value** |
| --- | --- | --- | --- | --- |
| **Setting type** |  |  |  |  |
| Outpatient | 980 (56%) | 386 (53%) | 594 (58%) | **0.04** |
| Emergency room | 759 (44%) | 336 (47%) | 423 (42%) |  |
|  |  |  |  |  |
| **Study type** |  |  |  |  |
| Industry-sponsored | 627 (36%) | 265 (37%) | 362 (36%) | 0.64 |
| Investigator-initiated | 1112 (64%) | 457 (63%) | 655 (64%) |  |

Data are presented as number of patients (%).

AF, atrial fibrillation.

**Table S7. Reported women frequencies and PPR values of previous studies assessing women representation in AF trials**

| **Study** | **Women frequency** | **Women PPR** |
| --- | --- | --- |
| Eshera *et al.*, 2015 | Undisclosed | < 1.2 |
| Scott *et al.*, 2018 | 37.8% | 0.8 – 1.1 |
| Cho *et al.*, 2021 | 14% – 40% | 0.8 |
| Khan *et al.*, 2021 | 35.2% | 1.00 |
| Alipour *et al.*, 2022 | 39.1% | 0.81 ± 0.28 |

AF, atrial fibrillation; PPR, Participation to Prevalence Ratio.
